# Practical Cystoscopy and the Diagnosis of Surgical Diseases of the Kidneys and Urinary Bladder

**Published:** 1911-09

**Authors:** 


### BOOKS AND AUTHORS

**Practical Cystoscopy and the Diagnosis of Surgical Diseases of the Kidneys and Urinary Bladder.** By **Paul M. Pilcher,** M. D., Consulting Surgeon to the Eastern Long Island Hospital. Octavo of 398 pages, with 233 illustrations, 29 in colors. Philadelphia and London: W. B. Saunders Company. 1911. (Cloth, $5.50 net.)

If one were to pick and choose from the relatively large number of books on genitourinary subjects there could be no mistake in selecting this as the most valuable contribution to the literature of the specialty. It is not alone complete as to construction and description, but it is absolutely flawless in the technic of bladder instrumentation and ureteral catheterisation. Dr. Pilcher has that rare gift of imparting knowledge by printed word and so excellent is his phraseology that it is possible for a careful student of previous casual experience with the cystoscope to do satisfactory work with the instrument by following the rules laid down in this most refreshing work. One of the chief reasons for this is that the teachings contained in the book are based upon the personal experience of the author; and, while he has undoubtedly been influenced by the writings of others nothing has been set down in this cystoscopy that he personally has not seen and tested. He makes grateful acknowledgement of the influence of the men under whom he has studied and of a truth their methods are traceable in Pilcher's work for he has the authority of Nitze and the directness and force of description of Caspar. The architecture of the book is beyond criticism.

Under the general heading, the technic of cystoscopy, types and constructions of American and European instruments and their lighting arrangements are described in detail and the direct and indirect instruments are differentiated. Considerable space is given to the care of the instrument, a necessary section which has been slighted in previous works. There is detailed instructions regarding the preparation of patients, the method of conducting an examination and for observing and orienting the urinary bladder. With this solid foundation the author takes his reader on to the next step, the catheterisation of the ureters with the various instruments devised for this work. The diagnostic value of ureteral catheterism is given a chapter by itself. Then are taken up, the diseased bladder with its pathologic change, the cystites, tuberculosis, ulcers, tumors and calculi and the various miscellaneous diseases not generally recognised. A section of the book is devoted to diseases of the prostate which lacks only that important and frequent condition resulting from colon infection of the prostatic urethra to make it complete. Future editions of this work will undoubtedly contain this phase of prostatic disease for while it is definitely urethral when it makes itself manifest there are few such cases in which the cystoscopic picture is not of value in a confirmatory sense at least.

The diseases of the ureter are given ample space and to the functional activity of the kidneys is given a separate part of the book. The diseases of the kidney are completely gone into and there is little that anyone however carping and critical could find to disagree with. The book closes with a chapter on the therapeutic uses of the cystoscope which is highly instructive.

There are over 200 illustrations, 29 of them being in color and these deserve especial mention. They are the work of Miss Eleanora Frye who made a special study of cystoscopy for the purpose of carrying out the illustrative portion of the work. It is unnecessary to say that she has been eminently successful in reproducing actual conditions in perfect color scheme. Her color plates are exquisite and add materially to the value of the book.

